# LncRNA H19 regulates smooth muscle cell functions and participates in the development of aortic dissection through sponging miR-193b-3p

**DOI:** 10.1042/BSR20202298

**Published:** 2021-01-22

**Authors:** Mingming Ren, Tao Wang, Xiaolong Wei, Yizeng Wang, Chun Ouyang, Yilian Xie, Xiaoqiang Ye, Zhen Han

**Affiliations:** 1 Department of Cardiovascular Surgery, Peking University Shenzhen Hospital, 1120 Lianhua Road, Shenzhen City, Guangdong Province, PR China; 2 Department of Vascular Surgery, Shanghai Changhai Hospital, Shanghai, PR China; 3 Department of Surgery, School of Medicine, Shantou University, Shantou, PR China

**Keywords:** aortic dissection, differentiation, LncRNA H19, miR-193b-3p, smooth muscle cell

## Abstract

**Background:** Multiple studies showed that long-chain noncoding RNA H19 (LncRNA H19) is high-expressed in human and mouse abdominal aortic aneurysms (AAAs). We speculated that it plays an important role in arterial disease, and therefore studied the role and mechanism of H19 in aortic dissection (AD).

**Methods:** The expressions of related genes in human aortic smooth muscle cells (HASMCs) induced by platelet-derived growth factor BB (PDGF-BB) or in the aortic tissue of AD patients/mice were identified by Western blot and quantitative real-time polymerase chain reaction. The targeting relationship between H19 and miR-193b-3p was predicted and verified by bioinformatics analysis, dual luciferase assay, RNA pull-down assay, RNA immunoprecipitation (RIP), and Pearson correlation coefficient. The H19 and miR-193b-3p effects on the biological functions of tissues and cells were examined by MTT (3-(4,5-dimethyl-2-thiazolyl)-2,5-diphenyl-2-H-tetrazolium bromide, thiazolyl blue tetrazolium bromide) assay, wound-healing assay, and Hematoxylin–Eosin (HE) staining.

**Results:** LncRNA H19 was abnormally high-expressed in thoracic aorta tissues of AD patients, and it could competitively bind to and inhibit miR-193b-3p. In the PDGF-BB group, the expressions of H19, matrix metallopeptidase (MMP) 2 (MMP-2) and MMP-9 were up-regulated and the expressions of miR-193b-3p, α-SMA, and SM22α were down-regulated; moreover, the proliferation and migration rate of HASMCs were increased. However, H19 silencing reversed the regulation of PDGF-BB on HASMCs. More interestingly, miR-193b-3p inhibitor could partially reverse the effect of H19 silencing. In addition, the above results were verified by animal experiments, showing that shH19 and up-regulated miR-193b-3p could significantly reduce the thoracic aorta pathological damage in AD mice.

**Conclusion:** LncRNA H19 regulated smooth muscle cell function by sponging miR-193b-3p and it participated in the development of AD.

## Introduction

Aortic rupture/dissection (AD) is caused by an intima-media tear in the aorta under the impact of high velocity and pressure blood flow, forming a false or true lumen [1]. AD has a high mortality rate and a poor prognosis, and is a clinically urgent problem to be solved [2]. Thoracic AD (TAD) is a type of AD, according to pathological morphology [2]. Recent studies found that TAD is a comprehensive pathological change process caused by pathological changes involving multiple blood vessel constituents, such as human aortic smooth muscle cells (HASMCs) and extracellular matrix (ECM) [3,4].

In the pathogenesis of TAD, vascular smooth muscle cells (VSMCs) play an important role in the process of aortic wall contraction and synthesis in the presence of the stimulation of various cells to promote vascular remodeling [5]. With the phenotypic change, VSMCs transform from contraction (differentiation) phenotype to synthesis (dedifferentiation) phenotype [6,7]. Dedifferentiated VSMCs showed higher viability in terms of proliferation, migration and synthesis, and at the same time, the expressions of differentiation markers α-SMA and SM22α were down-regulated [6]. In addition, ECM is the main component that forms the morphology of the aortic blood vessel wall, and in the vascular wall tissues of TAD patients, ECM shows obvious abnormalities [8]. Although a large number of studies demonstrated that some factors such as matrix metalloproteinases (MMPs) directly participate in the degradation of the ECM of aorta [9], but the interaction of these factors and their upstream regulatory factors are still not clear.

Abnormal expressions or functions of long-chain noncoding RNAs (lncRNAs) are closely related to many diseases such as cardiovascular diseases, cancers, and neurodegenerative diseases [10–12]. Study found that lncRNA H19 (H19) may play a mediating role between c-Myc and downstream gene expressions in colon cancer [13], and is up-regulated in liver cancer [14], bladder cancer [15] and breast cancer [16], suggesting that H19 may be related to the occurrence of cancer. Moreover, H19 also plays an important role in the network structure of multiple gene expressions in the human body [17]. For example, Wang et al. found that BRG1 interacts with lncRNA HIF1α-AS1, and that both may play an important role in the pathogenesis of TAD by regulating MMP-2/-9 expression level, apoptosis of VSMCs and phenotype conversion [18]. But this may not be the only mechanism for the development of TAD. The expression of H19 is significantly higher in the abdominal aorta samples of mouse abdominal aortic aneurysm (AAA) model than in normal aortic tissues [19,20]. Such a finding indicated that H19 may have a certain correlation with the AD process. LncRNAs, as an miRNA host transcript, regulate mRNA stability, and participate in intracellular life processes [21].

However, it is unclear whether the specific molecular mechanism of H19 in AD was consistent with the previously reported pathway. Therefore, the present study combined previous research to discuss the role and molecular mechanism of H19 in AD through clinical experiments, cell experiments, animal experiments, and other aspects.

## Materials and methods

### Tissues, cells, and infection

Thoracic aortic tissue of 25 patients with AD were collected during the operation, and 15 normal aortic samples were collected from age-sex matched patients undergoing valve replacement (August 2019 to December 2019).

HASMCs (PCS-100-012) were purchased from ATCC (U.S.A.). HASMCs were divided into the following four groups for research: Control, platelet-derived growth factor BB (PDGF-BB), PDGF-BB + shNC, and PDGF-BB + shH19. In the latter three groups, PDGF-BB (20 ng/ml) was used to treat HASMCs for 12 h, based on previous reports [22,23]. The cells were cultured in a 37°C and 5% CO_2_ incubator in DMEM/F-12 medium (11320033, Gibco, U.S.A.). The shNC lentiviral and shH19 lentiviral (pLKO.1, Target Sequence: CCCGTCCCTTCTGAATTTAAT) were constructed and packaged by Geneseed Biotech Co., Ltd. (CA). The cells were plated in 24-well plates at a density of 1 × 10^5^/well. When the degree of cell fusion reached 70–80%, the fresh culture medium containing 10 μg/ml polybrene was used to replace the original medium, and the cells were added with appropriate amount of virus suspension (shNC or shH19) and incubated at 37°C. To verify the effect of miR-193b-3p on the shH19 in ameliorating PDGF-BB-induced HASMCs, we divided HASMCs into the following groups: PDGF-BB, PDGF-BB + miR-193b-3p inhibitor control (IC), PDGF-BB+shH19 + IC, PDGF-BB+shH19+inhibitor (I). IC (miR2N0000001-1-5) and I (miR-193b-3p) were purchased from Ribobio (China).

### AD animal model construction

As previously described [24], Apolipoprotein E-deficient male mice (6–8 weeks old, Shanghai SLAC Company, CA) were used to construct AD animal models. All animals were randomly divided into the following four groups (*n*=12): Sham, AD, AD + shNC, AD + shH19. The mice were housed in specific SPF animal houses (12-h light/12-h dark) in Peking University Shenzhen Hospital. During 28 days of model construction, the mice were anesthetized with 3% isoflurane (Y0000858, Sigma–Aldrich, Germany). A subcutaneous osmotic micropump containing Ang II solution was implanted into the back of the mice in prone position through a small incision and closed with sutures (the flow rate was 1000 ng/kg/min). The mice in the AD + shNC and AD + shH19 groups received tail vein injection of lentivirus (1 × 10^11^ pfu/mouse) carrying shNC and shH19, followed by subcutaneous osmotic micropump implantation. Sham mice received an equal volume of saline injection. AD + agomiRNA Control and AD + agomiR-193b-3p groups mice (*n*=12) were injected with 10 mg/kg agomiR-193b-3p or agomiRNA Control via tail vein injection once a week (for 3 weeks), and other operations were the same as above. At the end of the model construction, after anesthesia (Pentobarbital Sodium, 85 mg/kg, 57-33-0, Sigma, U.S.A., intraperitoneal injection), the mice were killed by cervical dislocation, the thoracic aorta was excised, and histochemical staining and expression analysis were performed.

### Real-time quantitative PCR

Total RNA was extracted using TRIzol reagent (15596018, Invitrogen, U.S.A.). Cytoplasmic & Nuclear RNA Purification Kit (NGB-21000, NorgenBiotek, Canada) was performed to extract nuclear and cytoplasmic RNA. To obtain the required cDNA, PrimeScript RT Master Reagent (RR047A, Takara, CA) was used here. The quantitative reverse transcription real-time polymerase chain reaction (qRT-PCR) was detected by qRT-PCR instrument (QuantStudio 5, ABI, U.S.A.) and Real-time fluorescence quantitative PCR TB Green kit (RR820A). The qRT-PCR conditions were as follows (40 cycles): pre-denaturation at 95°C for 10 min, denaturation at 95°C for 15 s, annealing at 60°C for 1 min. The U6 and GAPDH were served as controls. The results of the experiment were quantitatively analyzed using the 2^−ΔΔ*C*_t_^ method [25]. The primers were displayed in Table 1.

**Table 1:** All primer in the present study

ID	Forward sequence (5′–3′)	Reverse sequence (5′–3′)
**H19**	ATGAAAGGTGAGGGGCTTCC	CCTTCCAGAGCCGATTCCTG
**GAPDH**	GGAGCGAGATCCCTCCAAAAT	GGCTGTTGTCATACTTCTCATGG
**H19-m**	GAACAGAAGCATTCTAGGCTGG	TTCTAAGTGAATTACGGTGGGTG
**GAPDH-M**	TGGCCTTCCGTGTTCCTAC	GAGTTGCTGTTGAAGTCGCA
**U6**	CTCGCTTCGGCAGCACA	AACGCTTCACGAATTTGCGT
**miR-193b-3p**	AAAGTCCCGCTGTCGTATCC	GTATCCAGTGCGTGTCGTGG
**α-SMA**	AAAAGACAGCTACGTGGGTGA	GCCATGTTCTATCGGGTACTTC
**α-SMA-M**	CCCAGACATCAGGGAGTAATGG	TCTATCGGATACTTCAGCGTCA
**SM22α**	GAAACCCACCCTCTCAGTCAG	TTGGCCATGTCTGGGGAAAG
**SM22α-M**	CCAACAAGGGTCCATCCTACG	ATCTGGGCGGCCTACATCA
**MMP2**	GCATCCAGACTTCCTCAGGC	CCATTAGCGCCTCCATCGTAG
**MMP2-M**	ACCTGAACACTTTCTATGGCTG	CTTCCGCATGGTCTCGATG
**MMP9**	CGACGTCTTCCAGTACCGAG	TTGTATCCGGCAAACTGGCT
**MMP9-M**	GGACCCGAAGCGGACATTG	CGTCGTCGAAATGGGCATCT

### Bioinformatics analysis

The GEO database (https://www.ncbi.nlm.nih.gov/geo/) (GSE92427) was used to analyze the expression of related miRNA in the aortic dissection of AD patients and healthy controls. Identification of differentially expressed (DE)-miRNA was performed by Limmar package (Fold Change = −2.16, *P*=0.01597). starBase v2.0 software was used to predict the potential targeted binding sites of H19 and miR-193b-3p.

### Dual-luciferase reporter assay

Wild-type H19 sequence fragments (5′-UGGGGCCUGAGGCCAGU-3′) and mutant-type H19 sequence fragments (5′-UUAGGUCACGCUAUCUA-3′) were inserted into the pmirGLO vector (E1330, Promega, CA, U.S.A.) to form H19-WT and H19-MUT plasmids, respectively. HEK293T cells (CRL-11268, ATCC, U.S.A.) were simultaneously transfected with miR-193b-3p mimic and H19-WT or H19-MUT. After 48 h of plasmids transfection of each group, the medium was discarded and the dual-luciferase reaction intensity was measured in strict accordance with the kit instructions (FR201-01, TransGen Biotech, CA).

### RNA pull-down assay

According to the instructions of the RNA pull-down assay kit (11685597910, Pierce, Rockford, U.S.A.), the biotinylated RNA and the structure buffer were mixed at a ratio of 1:500. Afterward, the magnetic beads (95°C, 2 min; ice bath, 3 min) were resuspended. The magnetic bead–RNA complex was washed three times with 500 μl of washing solution, and added with cell lysate (10 μl). Subsequently, RNA pull-down washing solution (500 μl) was added, and cell lysate (10 μl) was added. After measuring the protein concentration using the BCA method, Western blot analysis was performed to determine protein expressions.

### RNA immunoprecipitation

The binding of H19 to argonaute 2 (AGO2) proteins was performed according to the instructions of the RNA immunoprecipitation (RIP) kit (RIP-12RXN, Merck Millipore, U.S.A.). Briefly, cell extracts were incubated with antibodies and magnetic beads at 4°C overnight. The magnetic bead antibody complex was resuspended in RIP-wash buffer (900 μl). To collect the magnetic bead binding protein complex, the sample was placed on a magnetic base. After separation by proteinase K, RNA was extracted from the sample and part of the cell extract sample, and then detected by PCR. RIP antibodies were AGO2 (ab32381, 1:50, Abcam, U.S.A.) and immunoglobulin G (IgG) (1: 100, ab109489, Abcam, U.S.A.).

### Detection of cell viability

The trypsin EDTA solution (0.25%, 25200-056, GIBCO, U.S.A.) was added to each group of HASMCs to prepare a cell suspension (1 × 10^4^/ml). The cell suspension (100 μl/well) was added to a 96-well culture plate, followed by routine incubation for 24, 48, and 72 h, respectively. Ten microliters of MTT (3-(4,5-dimethyl-2-thiazolyl)-2,5-diphenyl-2-H-tetrazolium bromide, thiazolyl blue tetrazolium bromide) reagent (CT02, Sigma–Aldrich, Germany) was added to the culture well to culture the cells for 4 h. After removing the MTT supernatant, 100 μl/well DMSO (ST038, Beyotime, U.S.A.) was added to the culture wells and shaken at low speed for 10 min. A microplate reader (1681130, Bio-Rad, U.S.A.) was used to measure the absorbance at 570 nm.

### Wound-healing assay

The concentration of HASMCs suspension was adjusted to 1 × 10^5^ cells/ml. The cell suspension of each group was routinely incubated for 24 h to achieve a monolayer cell fusion degree of 70–80%. A sterile pipette tip was used to draw a ‘1’ on the cell membrane. The culture medium was gently washed twice to remove the detached cells. Subsequently, fresh medium was added to each well and cultivation continued for 24 h. Microscope (BZ-8100, Keyence, Japan) and Image-Pro Plus 4.1 analysis software (Media Controlnetics Company, U.S.A.) were used for observation and image analysis.

### Western blotting assay

The expression of α-SMA, SM22α, MMP-2, MMP-9 in the HASMCs and aortic tissues were detected by western blot [26]. The RIPA method (P0013, Beyotime, CA) was used to extract the total protein from each group of HASMCs and aortic tissues, and the BCA method was used to determine the protein content. The protein was separated by SDS/PAGE and then transferred to the PVDF membrane (Immobilon-P Transfer Membrane, EMD Millipore Corporation, MA). The membrane was sealed in a box with 5% skimmed milk at room temperature for 2 h. Then the primary antibodies (α-SMA (ab5694, 42 kDa, 1 µg/ml, Abcam, U.K.), SM22α (ab14106, 23 kDa, 1 µg/ml), MMP-2 (ab92536, 74 kDa, 1/1000), MMP-9 (ab38898, 92 kDa, 1/1000), and GAPDH (ab181602, 36 kDa, 1/10000)) were added for overnight incubation in a refrigerator at 4°C. After washing three times with TBST, the corresponding secondary antibodies such as anti-Mouse IgG (1:5000, ab205719) or anti-Rabbit IgG (1:5000, ab205718) were added and incubated for 1 h. The chemiluminescence kit (SL1350: 100 ml, Coolaber, China) was performed for exposure. The results were displayed by relative expression of protein (gray value of target protein/gray value of GAPDH), and analyzed with ImageJ analysis software (version 5.0, Bio-Rad, U.S.A.).

### Hematoxylin–Eosin staining

Mouse thoracic aorta tissues were fixed with 4% paraformaldehyde (P0099, Beyotime, CA) and embedded into paraffin. The sections were routinely deparaffinized, and the staining experiment was performed according to the instructions of the Hematoxylin–Eosin (HE) kit (C0105, Beyotime, CA). After the slices were dehydrated, made transparent, and sealed, the histopathological changes of the thoracic aorta in each group were observed and photographed under the CKX53 microscope (Olympus, Japan).

### Statistical analysis

The correlation between H19 and miR-193b-3p expression was analyzed by Pearson correlation coefficient. Each experiment was repeated three times independently. The data were expressed as mean ± SD, followed by analysis with SPSS v21.0 software. Comparisons between two groups were performed by *t* test. Multiple groups were expressed by one-way ANOVA for comparison. *P*<0.05 was defined as statistically significant.

## Results

### LncRNA H19 was abnormally high-expressed in thoracic aorta tissues of AD patients, and could bind and inhibit miR-193b-3p

**Figure 1:**
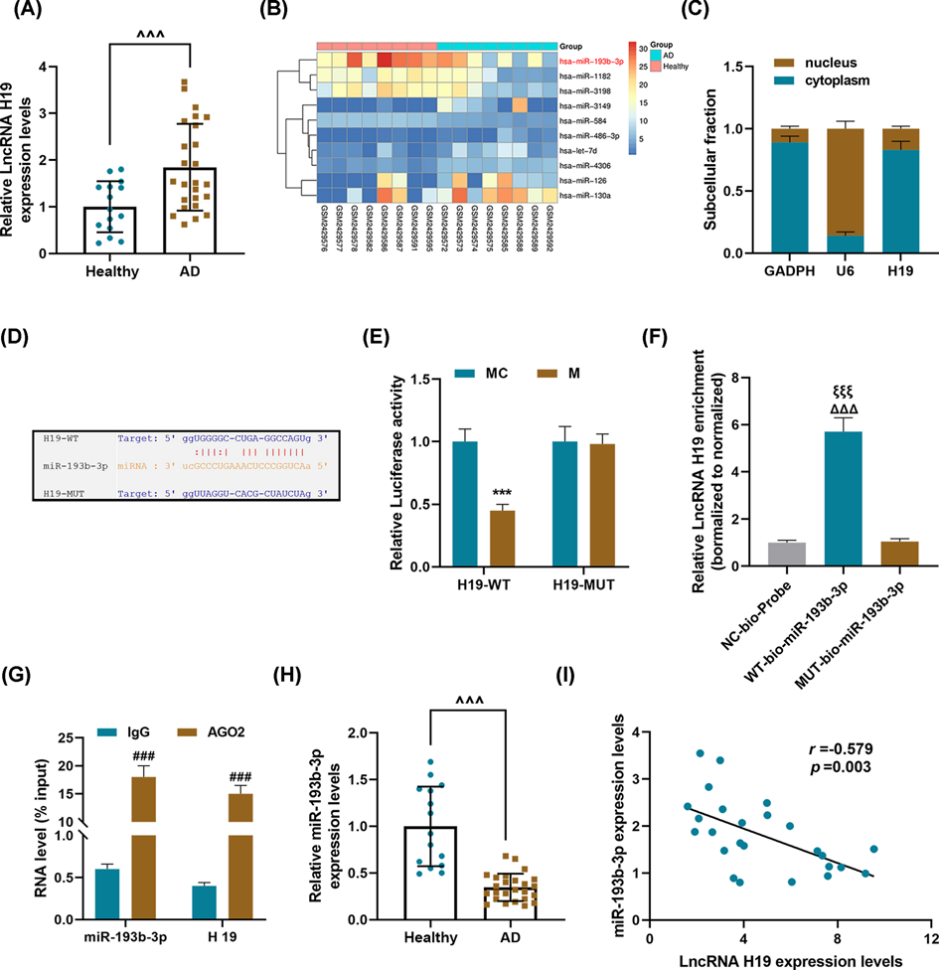
LncRNA H19 was abnormally high-expressed in thoracic aorta tissues of AD patients, and could competitively bind and inhibit miR-193b-3p (**A**) qRT-PCR was used to detect the expression of lncRNA H19 in the aorta of AD patients (*n*=25) and healthy aorta samples (*n*=15). GAPDH served as a control. Each sample was set to three replicate wells. (**B**) The GEO database (https://www.ncbi.nlm.nih.gov/geo/) was used to analyze related miRNA expression in AD. (**C**) The location of H19 in the HASMCs. GAPDH and U6 were used as internal reference genes. (**D**) The targeted binding site of lncRNA H19 and miR-193b-3p was predicted using starBase. (**E**) Dual-luciferase assay, (**F**) RNA pull-down assay, and (**G**) RIP experiments were used to verify the targeted binding relationship between lncRNA H19 and miR-193b-3p. (**H**) The expression level of miR-193b-3p in the sample tissues was detected by qRT-PCR. U6 serves as an internal reference gene. Each sample was set to six replicate wells. (**I**) The correlation between the expressions of lncRNA H19 and miR-193b-3p was analyzed using Pearson’s correlation coefficient. Each experiment was repeated three times independently at least, and the results were expressed as the means ± SD. ^^^^^*P*<0.001 vs. Healthy; ***P<0.001 vs. MC; ^###^*P*<0.001 vs. IgG; ^ΔΔΔ^*P*<0.001 vs. NC-bio-Probe; ^ξξξ^*P*<0.001 vs. MUT-bio-miR-193b-3p.

We determined the expression of H19 in the aorta samples of AD patients and healthy controls, and found that the expression of H19 in the AD group was significantly higher than that in the healthy group (*P*<0.001, Figure 1A). As shown in Figure 1B, analysis of GEO data (GSE92427) showed that miR-193b-3p was low-expressed in AD. Moreover, H19 was mainly expressed in cytoplasm (Figure 1C). The starBase database predicted that H19 could potentially target miR-193b-3p (Figure 1D). Dual-luciferase assay, RNA pull-down assay, and RIP were used for further verification (Figure 1E–G). The luciferase activity of miR-193b-3p mimic in H19-WT group was significantly lower than that of mimic control, but there was no difference in H19-MUT group (*P*<0.001, Figure 1E). As shown in Figure 1F,G, H19 was high-expressed in WT-bio-miR-193b-3p, and H19 and miR-193b-3p could bind to AGO2. These results suggested that H19 can competitively bind to miR-193b-3p via AGO2 (*P*<0.001). Therefore, we again determined the expression of miR-193b-3p in the AD aorta samples and normal aortic samples, and found that miR-193b-3p expression was significantly lower in the AD group than the healthy group (*P*<0.001, Figure 1H). Moreover, correlation analysis found that H19 was negatively correlated with miR-193b-3p (*r* = −0.579, *P*=0.003, Figure 1I).

### Effects of silencing lncRNA H19 on PDGF-BB-induced phenotypic differentiation and migration of HASMCs

**Figure 2:**
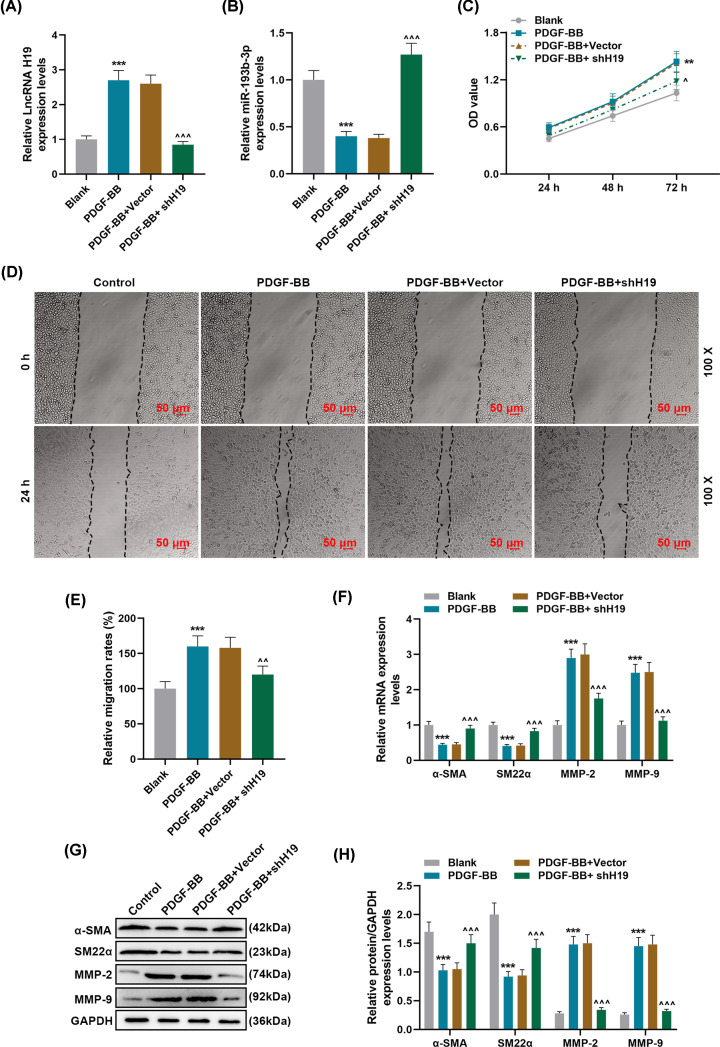
Effects of silencing lncRNA H19 on PDGF-BB-induced phenotypic differentiation and migration of HASMCs (**A,B**) The expression levels of lncRNA H19 and miR-193b-3p in Blank, PDGF-BB, PDGF-BB + Vector, PDGF-BB + shH19 groups were detected by qRT-PCR. GAPDH and U6 were used as internal reference genes. (**C**) After 24-, 48, and 72-h incubation of cells in each group, cell viability was detected by MTT method. (**D,E**) The cell migration of each group was detected by wound-healing assay. (**F**–**H**) qRT-PCR and Western blot were used to detect the levels of phenotypic differentiation markers α-SMA and SM22α, and the expressions of MMP-2 and MMP-9. GAPDH served as a control. Each experiment was repeated three times independently, and the results were the means ± SD. **P<0.01, ***P<0.001 vs. Blank; ^^^*P*<0.05, ^^^^*P*<0.01, ^^^^^*P*<0.001 vs. PDGF-BB + siVector.

To determine the role of H19 in AD, we built a PDGF-BB-induced HASMCs model and transfected shRNA H19 for further investigation. As shown in Figure 2A,B, PDGF-BB treatment significantly up-regulated the expression of H19, while significantly inhibiting the expression of miR-193b-3p (*P*<0.001). Compared with the PDGF-BB group, the expression of H19 in the PDGF-BB + shH19 group was significantly down-regulated, while the expression of miR-193b-3p was up-regulated (*P*<0.001, Figure 2A,B). As shown in Figure 2C–E, the results demonstrated that PDGF-BB treatment greatly promoted cell proliferation and migration, which were noticeably inhibited by H19 silencing (*P*<0.05). In addition, PDGF-BB treatment down-regulated the levels of differentiation markers α-SMA and SM22α, but compared with PDGF-BB group, PDGF-BB + shH19 group significantly up-regulated the levels of α-SMA and SM22α (*P*<0.001, Figure 2F–H, Supplementary Figure S2). PDGF-BB treatment obviously up-regulated the expressions of MMP-2 and MMP-9; H19 silencing significantly inhibited the promotion of PDGF-BB treatment on the expressions of MMP-2 and MMP-9 (*P*<0.001, Figure 2F–H).

### Effect of miR-193b-3p inhibitor on shH19 in reducing PDGF-BB-induced dedifferentiation and migration of HASMCs

**Figure 3:**
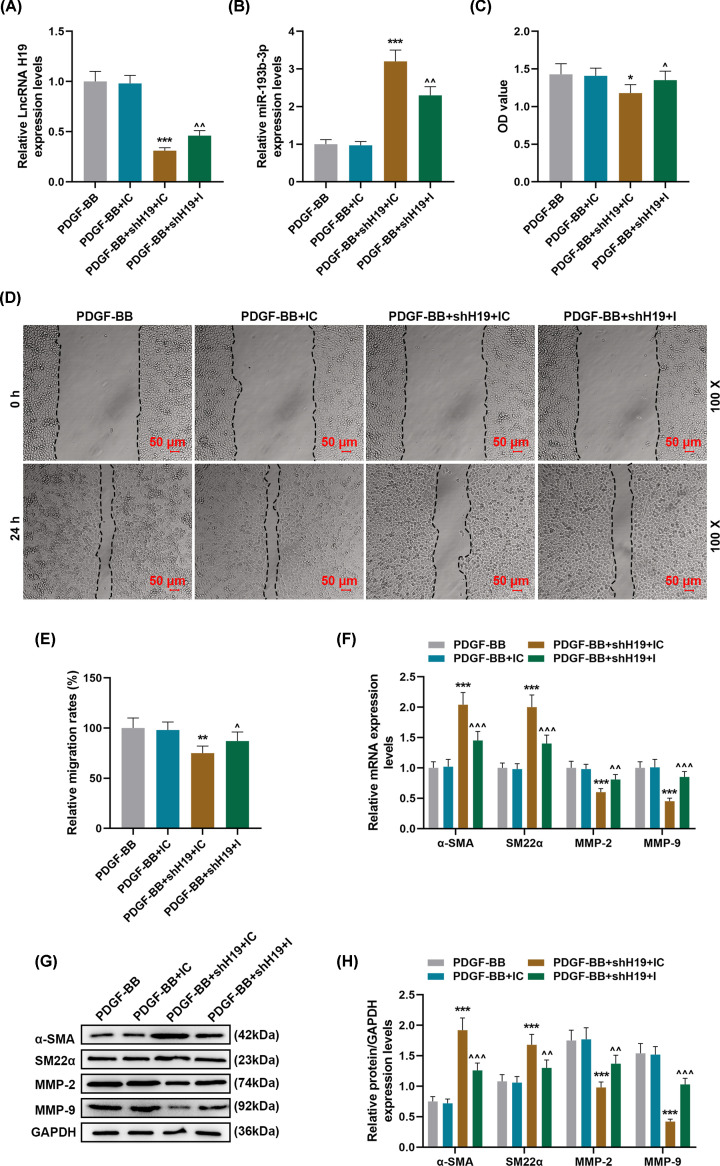
The effect of silencing miR-193b-3p on shH19 inhibiting PDGF-BB-induced dedifferentiation and migration of HASMCs (**A,B**) The expressions of lncRNA H19 and miR-193b-3p in PDGF-BB, PDGF-BB + IC, PDGF-BB + shH19 + IC, PDGF-BB + shH19 + I groups were detected by qRT-PCR. GAPDH and U6 were used as internal reference genes. (**C**) After 72 h of incubation, the cell viability was detected by MTT method. (**D,E**) The cell migration of each group was detected by wound-healing assay. (**F**–**H**) qRT-PCR and Western blot were used to detect the levels of phenotypic differentiation markers α-SMA and SM22α, as well as the expressions of MMP-2 and MMP-9. GAPDH served as a control. Each experiment was repeated three times independently, and the results were expressed by the means ± SD. I: miR-193b-3p inhibitor. *P<0.05, **P <0.01, *** *P*<0.001 vs. PDGF-BB + IC; ^^^*P*<0.05, ^^^^*P*<0.01, ^^^^^*P*<0.001 vs. PDGF-BB + shH19 + IC.

To determine the role of H19 and miR-193b-3p in cell biological functions, miR-193b-3p inhibitor was used in rescue experiments. Compared with PDGF-BB, the expression of H19 was significantly down-regulated in PDGF-BB + shH19 + IC group (*P*<0.001, Figure 3A). In addition, the expression of H19 in the PDGF-BB + shH19 + I group was significantly higher than that in the PDGF-BB + shH19 + IC group (*P*<0.01, Figure 3A). As shown in Figure 3B, miR-193b-3p inhibitor can greatly reverse the effect of shH19 on promoting the expression of miR-193b-3p in HASMCs (*P*<0.01). In addition, functional experiments showed that miR-193b-3p inhibitor can significantly reverse the inhibitory effect of shH19 on cell viability and migration ability in PDGF-BB-HASMCs group (*P*<0.05, Figure 3C–E). As expected, miR-193b-3p inhibitor can also reverse the regulatory effect of H19 silencing on the expressions of α-SMA, SM22α, MMP-2, and MMP-9 in PDGF-BB-HASMCs group (*P*<0.01, Figure 3F–H, Supplementary Figure S3).

### Effect of lncRNA H19 silencing and miR-193b-3p overexpression on AD animal models

**Figure 4:**
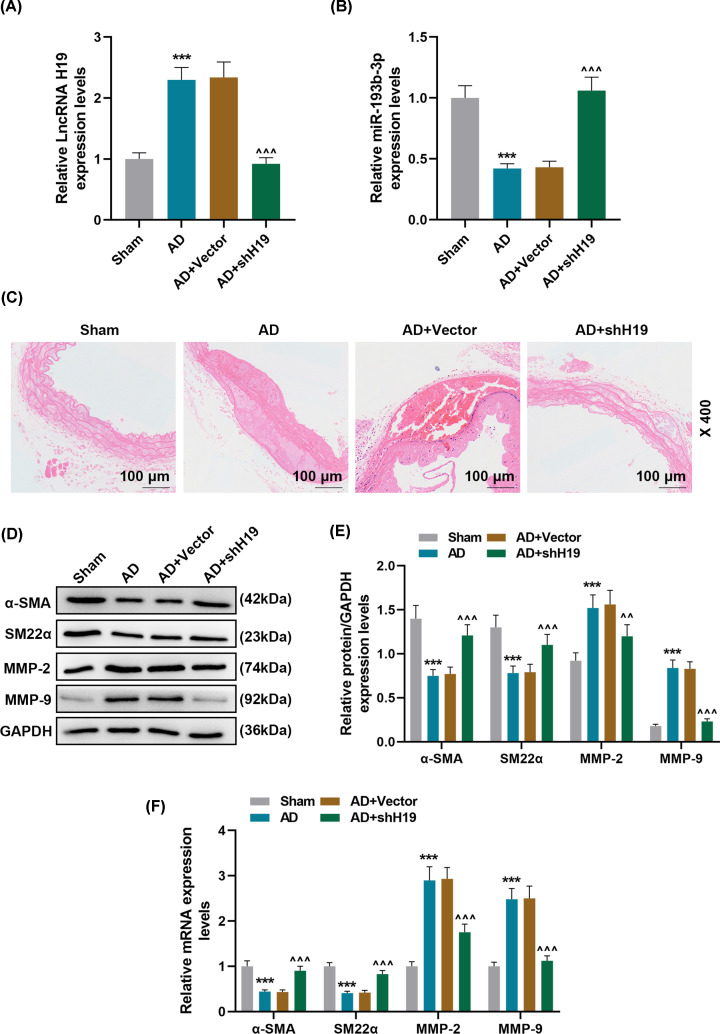
Effect of lncRNA H19 silencing on AD animal models (**A,B**) The expression levels of lncRNA H19 and miR-193b-3p in Sham, AD, AD + Vector, AD + shH19 groups were detected by qRT-PCR (*n*=6). GAPDH and U6 were used as internal reference genes. (**C**) The histopathological changes of each group were evaluated by HE staining (*n*=6). (**D**–**F**) qRT-PCR and Western blot were used to detect the levels of phenotypic differentiation markers α-SMA and SM22α and the expression of MMP-2 and MMP-9 in each group. GAPDH served as a control. Each experiment was repeated three times independently at least, and the results were the means ± SD. qRT-PCR: quantitative reverse transcription real time polymerase chain reaction. ***P<0.001 vs. Sham; ^^^^*P*<0.01, ^^^^^*P*<0.001 vs. AD + Vector.

**Figure 5:**
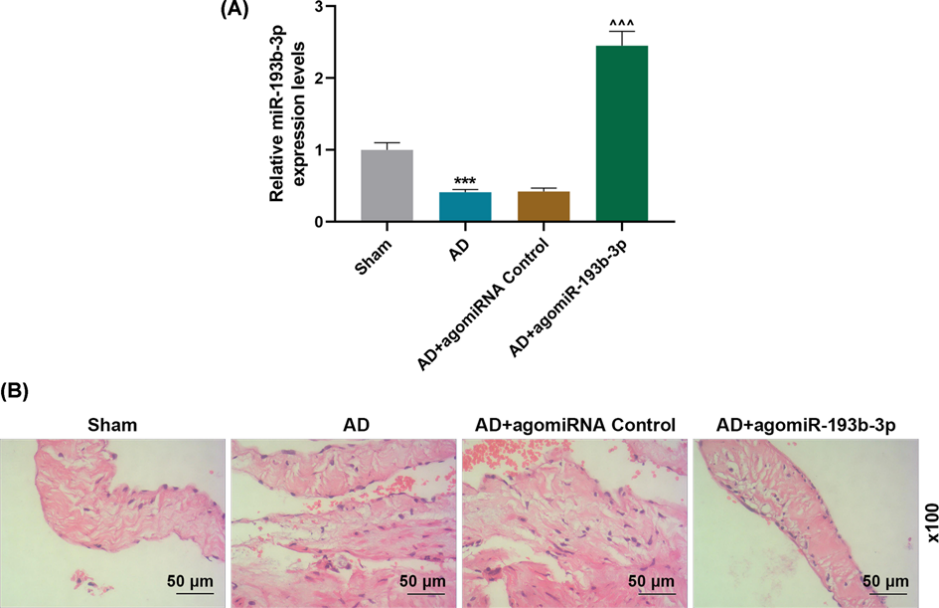
Effect of miR-193b-3p agomir on AD animal models (**A**) The expression levels of miR-193b-3p in the Sham, AD, AD + agomiRNA Control, and AD + agomiR-193b-3p groups were detected by qRT-PCR (*n*=6). U6 were used as internal reference genes. (**B**) The histopathological changes in each group were evaluated by HE staining (*n*=6). ***P<0.001 vs. Sham; ^^^^^*P*<0.001 vs. AD + agomiRNA Control.

AD animal model was used to verify the results of *in vitro* experiments. As shown in Figure 4A,B, compared with the Sham group, the expression of H19 in the AD group was significantly up-regulated, but that of miR-193b-3p was greatly down-regulated. Moreover, injection of AD mice with lentivirus carrying shH19 noticeably up-regulate the miR-193b-3p level (*P*<0.001). HE staining results showed obvious vascular media degeneration in the AD group, whose muscle fiber assembly was disordered, and the middle membrane thickness in the thoracic aorta increased remarkably (Figure 4C). However, shH19 treatment can significantly reduce vascular medial degeneration and muscle fiber assembly disorder, and decrease the middle membrane thickness in the thoracic aorta (Figure 4C). In addition, compared with the Sham group, the expressions of α-SMA and SM22α were down-regulated and the expressions of MMP-2 and MMP-9 were up-regulated in the AD group. However, shH19 treatment could significantly reverse the abnormal expressions of α-SMA, SM22α, MMP-2, and MMP-9 in AD group (*P*<0.01, Figure 4D–F, Supplementary Figure S4). To study whether AD mice was ameliorated after administration of miR-193b-3p agomir, RT-qPCR and HE staining were carried out. It was found that the expression of miR-193b-3p was up-regulated in AD + agomiR- miR-193b-3p group compared with AD + agomiRNA Control group (*P*<0.001, Figure 5A). AgomiR-193b-3p significantly reduced the degeneration of vascular media and disordered muscle fiber assembly caused by AD (Figure 5B).

## Discussion

Multiple studies showed that H19 is high-expressed in human and mouse AAAs, suggesting that H19 may play an important role in arterial disease [19,20]. However, the two researches were limited to animal studies, and the roles and connection of H19 and miR-193b-3p in AD was unclear. In the present study, we found that H19 expression was up-regulated and miR-193b-3p expression was down-regulated in AD patients and mouse thoracic aorta tissues. The above results have also been verified by cell experiments. In addition, H19 can competitively bind to miR-193b-3p *in vivo* and *in vitro*. H19 silencing inhibited PDGF-BB-induced phenotypic dedifferentiation and migration of HASMCs. Down-regulation of miR-193b-3p reversed the effect of shH19 on phenotypic differentiation and migration of HASMCs. *In vivo* experiments also proved that H19 silencing can reduce the histopathological damage of AD mice and promote the up-regulation of phenotypic differentiation markers and the down-regulation of MMP-2/9 in the tissues.

LncRNA plays a key role in regulating the occurrence and development of cardiovascular diseases, such as heart failure, aneurysm, AD, coronary heart disease, and some other diseases [11,16,17]. MRAK078262 negatively regulates CCRK to increase the rate of myocardial cell death and promote the development of heart failure [27]. H19 is low-expressed under normal circumstances, but some scholars have found that it is high-expressed in patients with atherosclerosis and increased risk of coronary heart disease [28]. A large number of studies confirmed that there is a mutual regulation relationship between lncRNAs and miRNAs that exert their physiological and pathological functions in various diseases through different regulatory forms [21,29,30]. It has been reported that lncRNA H19 promotes hepatocellular carcinoma metastasis and invasion by triggering and activating the miR-193b/MAPK1 axis [31]. Li et al. analyzed the development and progression of non-coding RNA in thoracic and AAA diseases, and pointed out the lack of study on the role of lncRNA in AAA [32]. The role of H19 in AAA has been previously mentioned. For example, H19 plays a pathogenic role in the formation of AAA through let-7a/IL-6 inflammation pathway [20]. In view of the complexity of the molecular regulatory network in diseases, this may not be the only regulatory pathway of H19. Our research associated H19 with miR-193b-3p for the first time. The current results showed that H19 competitively bound to and inhibited miR-193b-3p during AD development, suggesting that H19 acts as an adsorbent sponge for miR-193b-3p. Mechanistically, H19 can capture the target sequence of miR-193b-3p and isolate miR-193b-3p from its target mRNA, thereby regulating the function of related physiological pathology in AD.

PDGF-BB co-expression has been found to regulate VEGF-induced abnormal angiogenesis by regulating VEGF-R2 signaling and endothelial cell proliferation [22,23]. Our study also applied PDGF-BB co-expression to induce proliferation and migration and phenotypic dedifferentiation of HASMCs, and found that silencing H19 can reverse the effect of PDGF-BB on HASMCs. The current research results suggest that H19 was abnormally expressed in various malignant tumors and had proto-carcinogenic activity [33,34]. However, H19 has also been found to have cancer suppressing activity, which can inhibit the malignant proliferation, invasion, and metastasis of tumors, and the formation of tumor neovascularization [35,36]. The above studies indicate that the function of H19 may be different in different diseases. According to the literature, H19-derived miR-675 directly targets PTEN to stimulate VSMC proliferation, similarly, VSMC apoptosis is induced by H19 via HIF1α [37]. The present study found that silencing H19 through miR-193b-3p can inhibit PDGF-BB-induced cell proliferation and migration, and promote HASMCs phenotypic differentiation.

The main pathological change of AD is the tearing of the middle layer of blood vessels, and VSMCs are the main cellular components of the middle layer of the aorta [3,4]. Animal experiments showed that AD mice had obvious vascular media degeneration, muscle fiber assembly disorder, VSMCs became large and round. These changes are consistent with the pathological changes of AD. More importantly, H19 silencing and miR-193b-3p agomir can reduce the pathological damage of thoracic aorta in AD mice. α-SMA and SM22α are specific marker proteins of aortic smooth muscle cells, and are also commonly used to identify the contractile phenotype of VSMCs [23,38]. MMPs can degrade many components in ECM and basement membrane, and can destroy the connective tissues of the arterial wall [23]. Among them, MMP-2 and MMP-9 are the most closely related to occurrence and development with AD [39]. Increased viability of MMP-2 can cause arteries to dilate, and increased vitality of MMP-9 can cause rupture of aortic aneurysms [40]. Consistent with previous studies [18], in this study, both *in vivo* and *in vitro* experiments proved that H19 silencing further promoted up-regulation of α-SMA and SM22α, and down-regulated MMP-2 and MMP-9 expressions, suggesting that H19 may participate in the pathogenesis of AD by regulating the expressions of MMP-2/-9 and the proliferation, migration, and phenotype transformation of HASMCs.

In summary, our research indicated that H19, as an miR-193b-3p sponge, regulates smooth muscle cell function and participates in AD vascular remodeling. Importantly, for the first time, we discovered that H19/miR-193b-3p is a new pathogenic pathway in AD. This provides a direction for further research on the role of H19 in AD and new theoretical support for the pathological mechanism of AD.

## Supplementary Material

Supplementary Figures S1-S15

## Data Availability

The analyzed datasets generated during the study are available from the corresponding author on reasonable request.

## Abbreviations

AAA: abdominal aortic aneurysm
AD: aortic dissection
AGO2: argonaute 2
α-SMA: alpha-smooth muscle actin
DMEM/F12: Dulbecco's Modified Eagle Medium /nutrient mixture F12
ECM: extracellular matrix
GAPDH: glyceraldehyde-3 phosphate dehydrogenase
GEO: Gene Expression Omnibu Altered long noncoding RNA expression profiles in the myocardium of rats with ischemic heart failure.
HASMC: human aortic smooth muscle cell
HE: Hematoxylin–Eosin
HIF1α-AS1: Hypoxiainducible factor1α antisense RNA 1
IC: inhibitor control
IgG: immunoglobulin G
lncRNA: long-chain noncoding RNA
MMP: matrix metallopeptidase/metalloproteinase
MTT: 3-(4,5-dimethyl-2-thiazolyl)-2,5-diphenyl-2-H-tetrazolium bromide, thiazolyl blue tetrazolium bromide
PDGF-BB: platelet-derived growth factor BB
qRT-PCR: quantitative reverse transcription real-time polymerase chain reaction
RIP: RNA immunoprecipitation
SM22α: Smooth muscle22 alpha
SPF: Specific pathogen free
TAD: thoracic AD
VEGF: vascular endothelial growth factor
VSMC: vascular smooth muscle cell
