# Supplementary figures and images for: LncRNA H19 regulates smooth muscle cell functions and participates in the development of aortic dissection through sponging miR-193b-3p
Source: Biosci Rep. 2021 Jan 22;41(1):BSR20202298. doi: 10.1042/BSR20202298 (PMC7823186; doi:10.1042/BSR20202298)

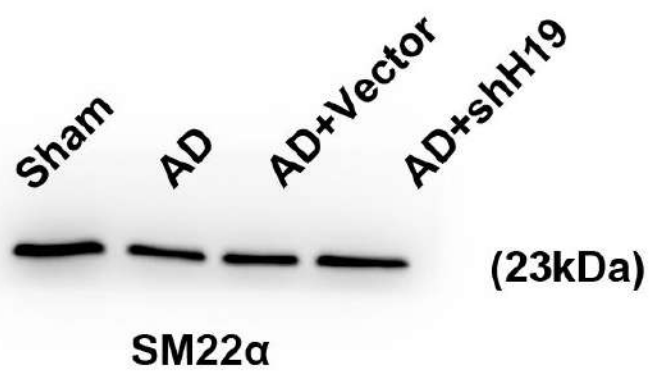

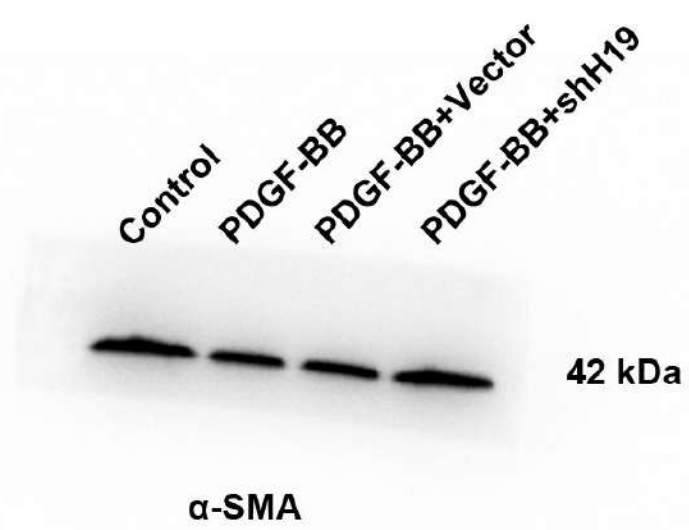

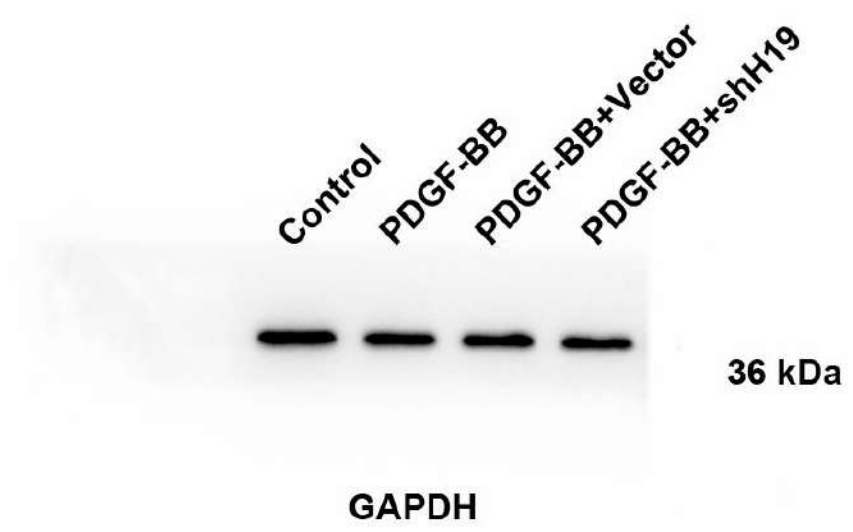

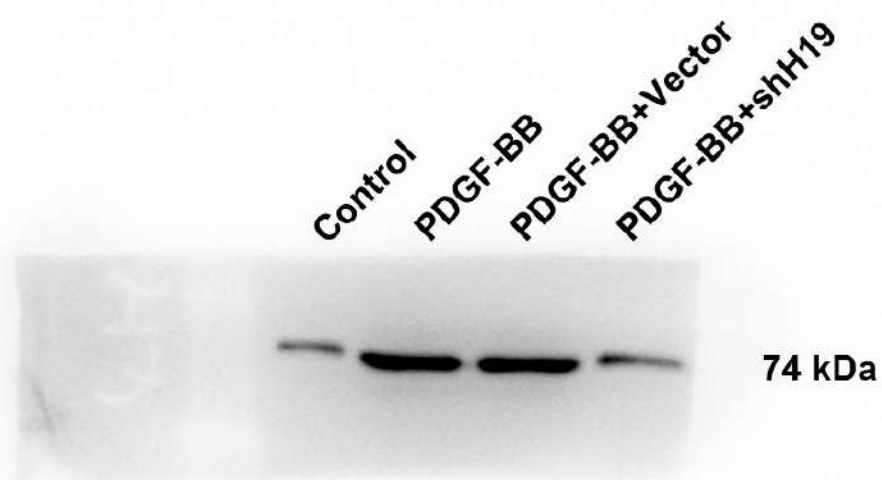

**MMP-2**

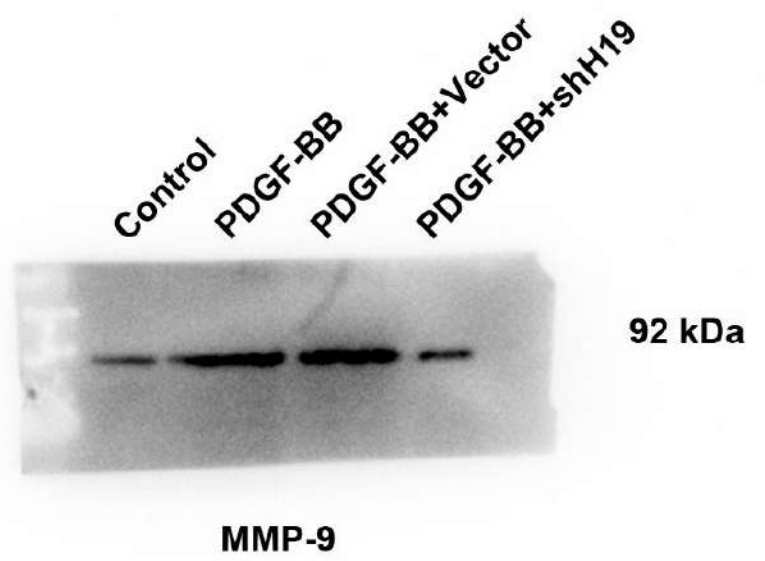

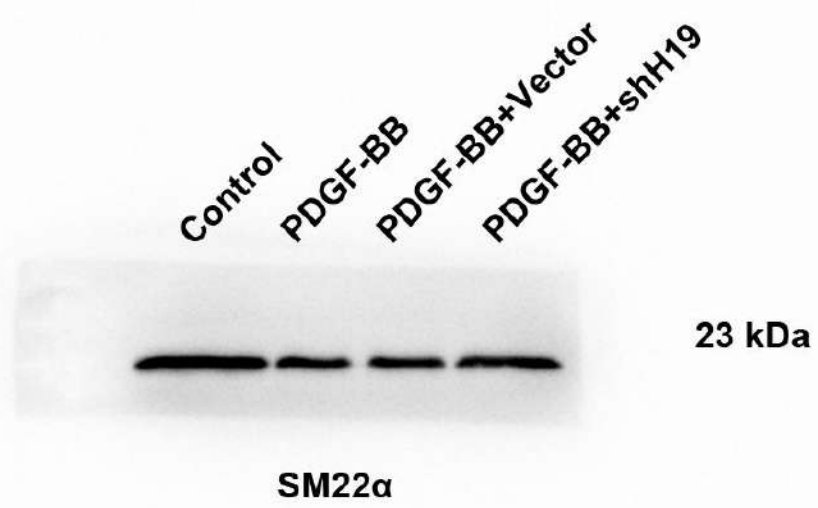

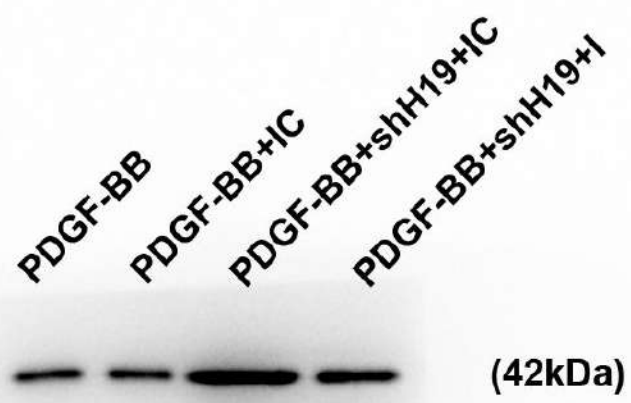

α-SMA

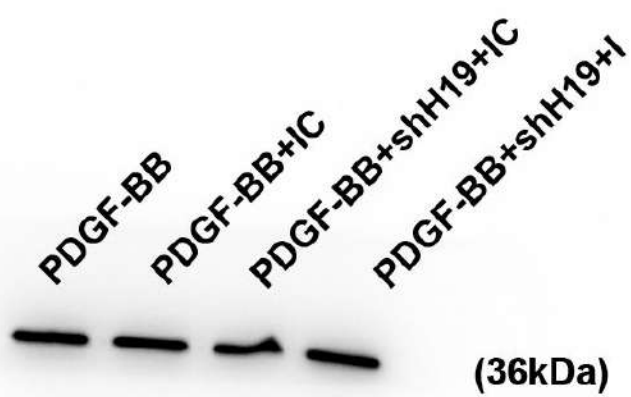

GAPDH

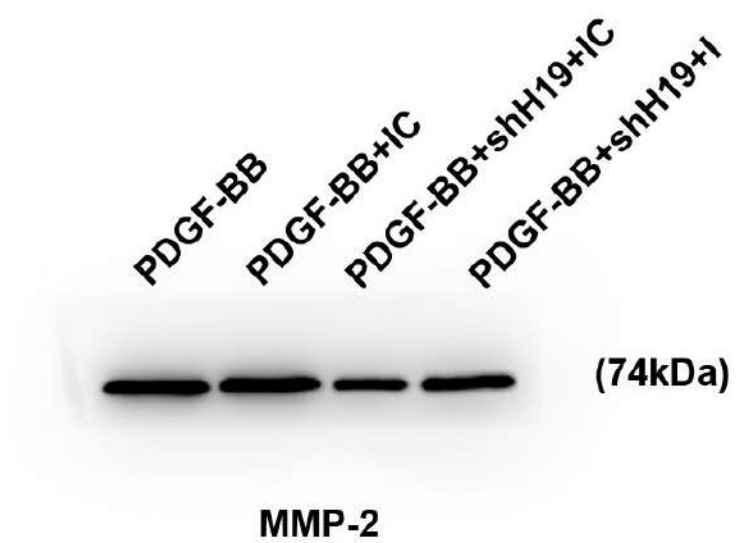

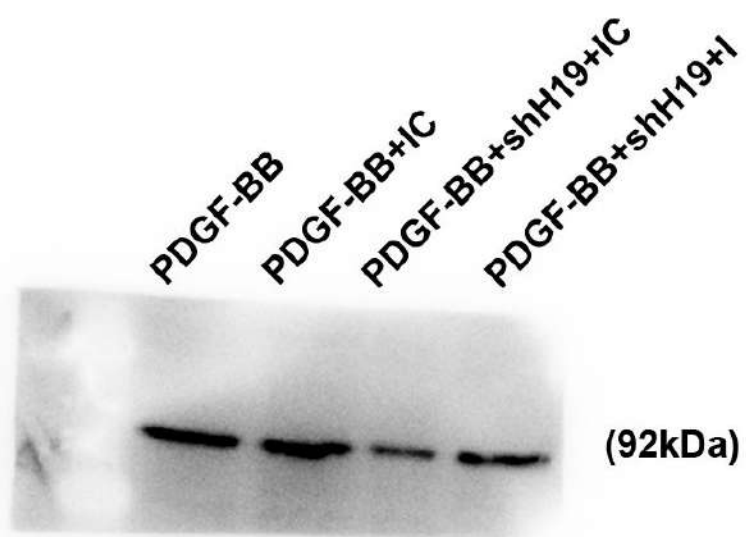

**MMP-9**

PDGF-BB  
PDGF-BB+IC  
PDGF-BB+shH19+IC  
PDGF-BB+shH19+I

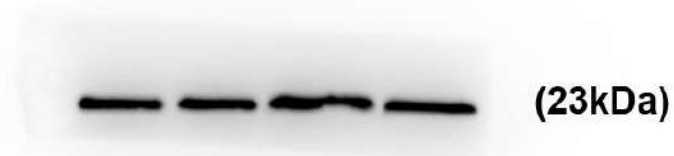

SM22 $\alpha$

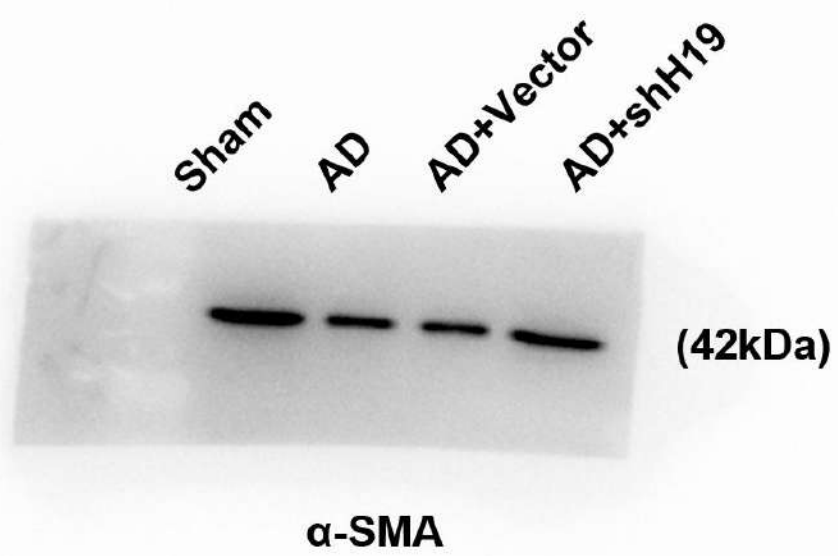

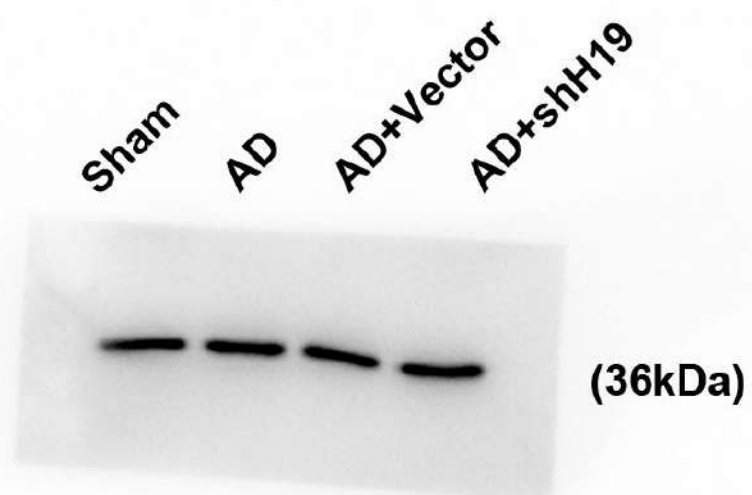

**GAPDH**

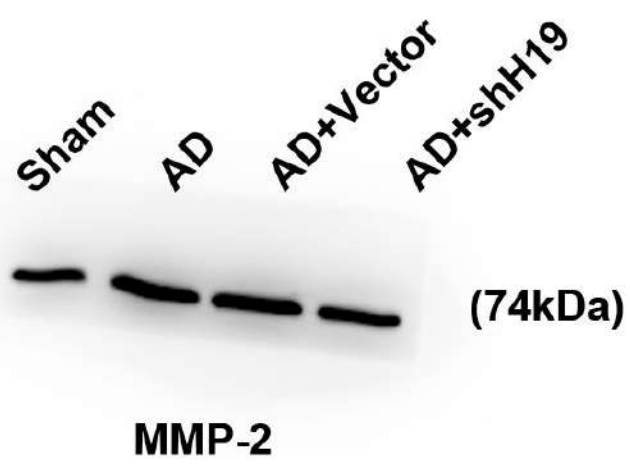

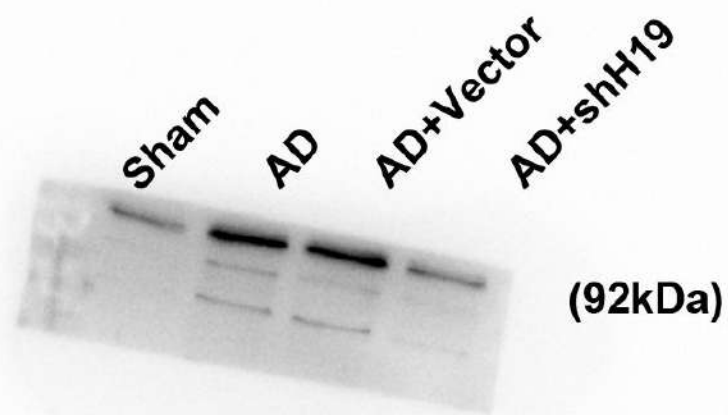

**MMP-9**

Supplement: Supplementary Figures S1-S15 [file BSR-2020-2298_supp.pdf]
